# Taking the train of digital health and artificial intelligence to improve medical laboratory service in Africa: Key considerations

**DOI:** 10.4102/ajlm.v12i1.2329

**Published:** 2023-11-30

**Authors:** Rajiv Erasmus, Pascale Ondoa

**Affiliations:** 1 Department of Chemical Pathology, Faculty of Medicine and Health Sciences, Stellenbosch University, Cape Town, South Africa; 2 Department of Pathology, Faculty of Medicine, University of Botswana, Gaborone, Botswana; 3 Department of Biomedical Sciences, Faculty of Health Sciences and Wellness, Cape Peninsula University of Technology, Cape Town, South Africa; 4 African Society for Laboratory Medicine, Addis Ababa, Ethiopia; 5 Department of Global Health, Institute of Global Health and Development, University of Amsterdam, Amsterdam, the Netherlands

Similar to other scientific fields, laboratory medicine is undergoing a digital revolution. Software, algorithms and digital applications are increasingly incorporated into in-vitro diagnostics to enhance automated processes for the collection, storage, integration and analysis of data to inform patient management, to aid in population-based investigations, or to improve the quality and effectiveness of laboratory testing.^1,2^ Some of the most popular examples of how digitisation can revolutionise the practice of laboratory medicine in Africa include automation of specimen management and testing (e.g., cobas^®^ system workflow from Roche), connection of various instruments to monitor their functionality in real time and assist operators (e.g., GeneXpert^®^ from Cepheid), and laboratory information systems that support the swift reporting of test results and real-time diagnostics of the performance of entire laboratory networks. Digital technologies are also being used to streamline processes for quality assurance and laboratory operations.^3^ Digitised laboratory testing processes provide new opportunities for uncovering trends, increase the usability of state-of-the-art technology and may accelerate the utilisation of test results for improved patient and public health outcomes.^3,4,5^

The emergence of digital data in the field of laboratory medicine brings the possibility of using artificial intelligence (AI) to aid in clinical decision-making, disease monitoring and prediction of outcomes and patient safety. Artificial intelligence is defined as the ability of computer systems to perform tasks that normally require human intelligence, such as learning, problem-solving, visual perception, decision-making and communication.^4,5,6,7,8^ Artificial intelligence takes advantage of digital data to integrate health records, symptom profiles, demographic data and other patient information to better predict diagnoses and recommend treatments. Machine learning is the type of AI most frequently used in laboratory medicine. One example of machine learning is a computer algorithm that can ‘digest’ historical information from thousands of pathology slides, identify patterns of interpretation and develop an algorithm for detecting cancer cells quickly through reducing human interpretation errors as well as the length of time required for visual inspection by a human operator.^4,5,6,7,8^ In another interesting use of AI, machine learning has been used in the prediction of the spread of coronavirus disease 2019, by analysis of viral variant data, hot spots and contact tracing.^2^ Artificial intelligence may also be able to assist in the development of new diagnostics and treatment strategies, which would be an important advance.^9^

The integration of digitisation and AI into laboratory medicine certainly has the potential to improve the accuracy, efficiency, usability and productivity of laboratory services. Indeed, the simultaneous scaling up of paper-based information, digitisation of information and processes, and implementation of AI are creating unique opportunities for leapfrogging from a conventional landline system to a digital diagnostic ecosystem. However, for African health systems that already face well-known chronic challenges, these changes also pose risks for overwhelming laboratory medicine with too many challenges or inadequate technology to meet them.^10^

Undoubtedly, integrating digitisation and AI into laboratory and diagnostic services implementation in the low- and middle-income settings of Africa is expected to pose some challenges and barriers. These include: (1) investment costs in required hardware, software, data storage and maintenance; (2) privacy, ethical and regulatory concerns around the sharing of sensitive personal data; and (3) human factors, including the building of computer and AI literacy among healthcare workers, as well as lack of trust lack of trust or confidence in digital technology and AI tools, or fear of losing jobs or skills.^11,12^ These challenges and barriers are amplified in many developing countries and may include issues around the standardisation of tools and the complexity of conducting quality assurance on digitised processes and the AI component of diagnostics. Lessons learnt from disruptions in settings already embracing digital technologies can inform the way Africa adopts and scales up AI into healthcare in general and diagnostics in particular.^11,12^

So how do we take it from there?

As with other technology innovations, the implementation of new digital technologies and AI should clearly respond to a clinical need and potential patient applications should be clearly defined by users of diagnostic services. The implementation of infrastructure should include multiple stakeholders that tap experts both within and outside of the health sectors. This is particularly important with respect to data acquisition, data creation, model development and deployment.

Critical to the scale-up and democratisation of digital technologies and AI is addressing gaps in access to electricity and Internet connections on the continent. As of September 2023, only 47% of Africans have reliable access to electricity (https://data.worldbank.org), and there was only 43% penetration for Internet access in 2021 (www.statista.com). Additionally, a whole cadre of experts must be built to manage data collection, storage, analysis and interpretation. This gap has already been demonstrated by the gap in bioinformaticians observed during the scale-up of next-generation sequencing in the context of the Africa Centres for Disease Control and Prevention Pathogen Genomic Initiative.^13^ Finally, a thorough monitoring and evaluation framework is warranted to assess where and how AI and digital technology are actually improving patient and public health and how these can be integrated and implemented in African healthcare settings.

Integrating digital technologies and AI into laboratory services has the potential to transform diagnostic services and contribute to the implementation of the May 2023 resolution of 76th World Health Assembly on strengthening diagnostics in Africa and other countries to improve positive patient, public and global health outcomes.^14^ Regional agencies such as the Africa Centres for Disease Control and Prevention have already taken the lead in developing continental frameworks to guide the implementation of digital technologies in public health systems,^15^ as well as in launching initiatives such as HealthConnekt to build the internet connectivity infrastructure in Africa, to allow the continent to align with the World Health Organization’s deliberate strategy to use digital technology as a force to transform primary healthcare.^16^ Moving forward, Africa-led knowledge networks, such as the African Society for Laboratory Medicine’s Laboratory Strengthening Community of Practice and Laboratory Directors Forum^17,18^ can play a major role in identifying priorities, best practices, operational requirements and ethical considerations for the implementation of digital and AI technology in African laboratory medicine.

The digital revolution is a game changer for Africa, which can drive a parallel digital revolution in laboratory diagnostics. Continental frameworks that have been developed now need to be made operational to benefit from this transformation.
